# Fission Yeast Mto1 Regulates Diversity of Cytoplasmic Microtubule Organizing Centers

**DOI:** 10.1016/j.cub.2010.10.006

**Published:** 2010-11-09

**Authors:** Itaru Samejima, Victoria J. Miller, Sergio A. Rincon, Kenneth E. Sawin

**Affiliations:** 1 Wellcome Trust Centre for Cell Biology, University of Edinburgh, Swann Building, Mayfield Road, Edinburgh EH9 3JR, UK

## Abstract

Microtubule nucleation by the γ-tubulin complex occurs primarily at centrosomes, but more diverse types of microtubule organizing centers (MTOCs) also exist, especially in differentiated cells [1–4]. Mechanisms generating MTOC diversity are poorly understood. Fission yeast *Schizosaccharomyces pombe* has multiple types of cytoplasmic MTOCs, and these vary through the cell cycle [5, 6]. Cytoplasmic microtubule nucleation in fission yeast depends on a complex of proteins Mto1 and Mto2 (Mto1/2), which localizes to MTOCs and interacts with the γ-tubulin complex [7–12]. Localization of Mto1 to prospective MTOC sites has been proposed as a key step in γ-tubulin complex recruitment and MTOC formation [9, 13], but how Mto1 localizes to such sites has not been investigated. Here we identify a short conserved C-terminal sequence in Mto1, termed MASC, important for targeting Mto1 to multiple distinct MTOCs. Different subregions of MASC target Mto1 to different MTOCs, and multimerization of MASC is important for efficient targeting. Mto1 targeting to the cell equator during division depends on direct interaction with unconventional type II myosin Myp2. Targeting to the spindle pole body during mitosis depends on Sid4 and Cdc11, components of the septation initiation network (SIN), but not on other SIN components.

## Highlights

► Conserved MASC motif in Mto1 contains multiple independent MTOC-targeting sequences ► Multimerization is important for MASC-dependent Mto1 localization ► Interaction with Myp2 controls Mto1 localization to cell equator in cell division ► Mto1 at mitotic spindle pole bodies requires Sid4 and Cdc11, but not other SIN proteins

## Results and Discussion

### Distinct Sequences within a Conserved Carboxy-Terminal Motif of Mto1 Are Required for Targeting to Interphase Spindle Pole Body, Mitotic Spindle Pole Body, and Cell Equator

Cytoplasmic microtubule organizing centers (MTOCs) in interphase fission yeast include the interphase spindle pole body (iSPB; yeast centrosome equivalent), as well as sites on the nuclear envelope and on microtubules (MTs) themselves [5, 6]. In mitosis, mitotic SPBs (mSPBs) act as MTOCs for cytoplasmic astral MTs during anaphase elongation of the intranuclear mitotic spindle. Later, during cytokinesis, equatorial MTOCs (eMTOCs) nucleate postanaphase arrays (PAAs) of MTs from the cell division site. Mto1 and its partner protein Mto2 are found at all of these MTOCs [7–12]. Mto1 is a large coiled-coil protein similar in overall structure to *Drosophila* centrosomin and to mammalian myomegalin and CDK5RAP2, which is mutated in a form of primary autosomal microcephaly [14–17]. All of these proteins share a conserved N-terminal sequence termed Centrosomin Motif 1 (CM1), which is implicated in γ-tubulin complex binding in several systems [7, 13, 14, 16].

**Figure 1:**
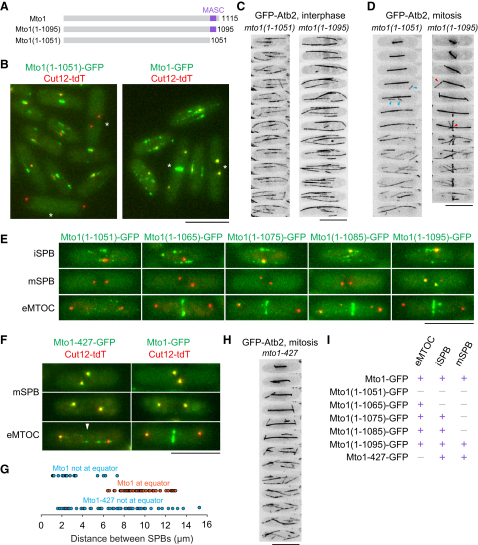
A Modular Sequence Motif in the Mto1 C Terminus Regulates Differential Localization to Multiple MTOCs (A) Position of conserved MASC motif in Mto1 and truncation mutants. (B) Cytoplasmic distribution of Mto1(1-1051)-GFP and Mto1-GFP (green) and SPB marker Cut12-tdTomato (tdT; red). Mitotic cells are marked with asterisks. (C and D) Time-lapse images of GFP-tubulin in interphase and mitotic *mto1(1-1051)-GFP* and *mto1(1-1095)-GFP* cells (2 min intervals). GFP signal from Mto1 itself is too faint to be seen here. Note mitotic astral and postanaphase array (PAA) MTs (red arrowheads) in *mto1(1-1095)-GFP* but random cortical MT nucleation in *mto1(1-1051)-GFP* cells (blue arrowheads). (E) Localization or absence of indicated Mto1-GFP truncations (green) at interphase SPBs (iSPB), mitotic SPBs (mSPB), and equatorial microtubule organizing centers (eMTOC). SPB marker Sad1-dsRed is in red. (F and G) Triple-point mutant Mto1-427-GFP (green) is present at mSPBs in metaphase and anaphase but absent from eMTOCs (white arrowhead). (G) Wild-type Mto1-GFP appears at eMTOCs as spindles elongate. (H) Time-lapse images of GFP-tubulin in mitotic *mto1-427* cells (2 min intervals). PAA MTs are absent. (I) Localization summary of mutant Mto1 proteins. Additional data are shown in Figure S1. Scale bars represent 10 μm.

We identified an ∼44 amino acid (aa) motif near the Mto1 C terminus (aa 1052–1095) that is conserved in the C terminus of a single protein in each of several fungal proteomes (Figure 1A; see also Figure S1A available online). Many of the proteins identified are likely orthologs of Mto1, because they contain N-terminal CM1 sequences and extensive regions of predicted coiled coil. The most distantly related C-terminal motif was in the budding yeast protein Spc72p. Spc72p lacks CM1 but is required for cytoplasmic MT nucleation from the SPB, the sole MTOC in budding yeast [18]. Because Spc72p functions analogously to Mto1, we refer to the conserved motif as MASC (*M*to1 *a*nd *S*pc72p *C* terminus).

A truncated Mto1 protein lacking MASC, Mto1(1-1051)-GFP, was absent from SPBs and eMTOC sites, unlike wild-type Mto1-GFP and Mto1(1-1095)-GFP, which both contain MASC (Figures 1B and 1E; Figure S1B) [7, 8]. GFP-tubulin imaging revealed an absence of astral and PAA MTs in *mto1(1-1051)-GFP* cells compared to *mto1(1-1095)-GFP* (Figure 1D; Movie S1; Movie S2; in GFP-tubulin imaging experiments, Mto1-GFP proteins are too faint to be seen against GFP-tubulin, but tagging with GFP was necessary to maintain levels of truncated Mto1 proteins; see Figure S1C). Instead of PAA nucleation, *mto1(1-1051)-GFP* cells showed sporadic, spatially random MT nucleation (Figure 1D; Movie S1), suggesting that temporal cell-cycle control of cytoplasmic MT nucleation is intact in *mto1(1-1051)-GFP* cells but that spatial control is not. Consistent with this, *mto1(1-1051)-GFP* cells were not impaired in broader cytoplasmic MT nucleation (Figure 1C; Figure S1J). Intranuclear mitotic spindle formation and elongation were normal in *mto1(1-1051)-GFP* cells, as is also the case for *mto1*Δ cells (Figure 1D) [7, 12].

We generated additional strains *mto1(1-1065)-GFP*, *mto1(1-1075)-GFP*, and *mto1(1-1085)-GFP*, which contain incremental subregions of MASC relative to *mto1(1-1051)-GFP*. Strikingly, these strains showed incremental restoration of Mto1 localization. Mto1(1-1065)-GFP was present at eMTOC sites but absent from SPBs, whereas Mto1(1-1075)-GFP and Mto1(1-1085)-GFP were present at eMTOC sites and iSPBs but absent from mSPBs (Figure 1E; Figures S1D–S1F). GFP-tubulin imaging showed that MT nucleation in these strains was again correlated with Mto1 localization, both in interphase and mitosis (Figures S1G–S1I). Consistent with microtubule nucleation phenotypes, RFP-tagged Alp4 (γ-tubulin complex subunit) colocalized with Mto1(1-1065)-GFP at eMTOC sites but was not observed at eMTOC sites in *mto1(1-1051)-GFP* cells (data not shown; see Supplemental Experimental Procedures).

The *mto1(1-1065)-GFP* strain demonstrates that Mto1 can localize to eMTOC sites independently of targeting to SPBs. To determine whether Mto1 can localize to SPBs independently of eMTOCs, we generated a series of single- and multiple-point mutations within the portion of MASC required for eMTOC localization in the context of full-length Mto1. A triple-point mutant (R1056A, E1059A, E1061A), termed Mto1-427, localized to iSPBs and mSPBs, but not to eMTOC sites (Figures 1F and 1G; additional data not shown), and *mto1-427* cells nucleated astral MTs in mitosis, but not PAA MTs (Figure 1H).

Collectively, these results demonstrate that different mechanisms and subregions of MASC regulate Mto1 localization to different subcellular sites—iSPBs, mSPBs, and eMTOCs–and that targeting to SPBs and eMTOC sites can occur independently of each other (Figure 1I). Moreover, the close correlation between Mto1 localization and MT nucleation sites in the mutants supports the earlier proposal that local recruitment of the γ-tubulin complex by Mto1/2 complex to specific intracellular sites converts prospective MTOCs into active MTOCs [9].

### Efficient MASC-Dependent Localization Requires Mto1 Multimerization

**Figure 2:**
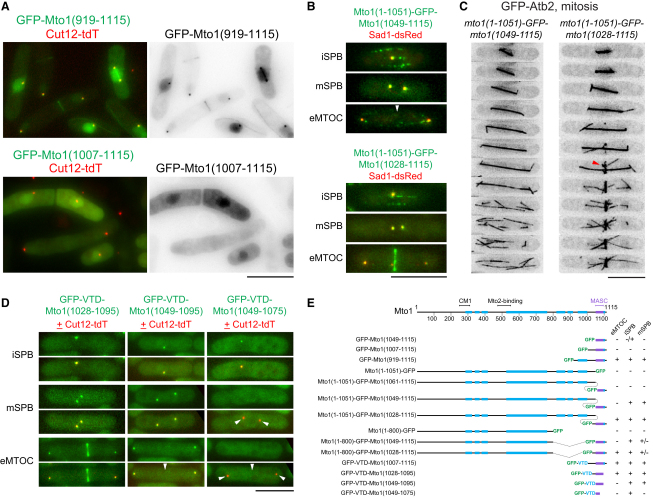
Multimerization of the Mto1 C Terminus Is Critical for Robust SPB and eMTOC Localization (A) Localization of indicated Mto1 C-terminal fragments fused to GFP. SPB marker Cut12-tdT is shown in red. (B) Mto1 localization to SPBs but not eMTOCs (arrowhead, top) and to SPBs and eMTOCs (bottom) in the indicated “Mto1 GFP-insertion” strains. SPB marker Sad1-dsRed is shown in red. (C) Time-lapse images (2 min intervals) of GFP-tubulin showing astral MT nucleation (left) and astral and PAA MT nucleation (right) in the Mto1 GFP-insertion strains. Red arrowhead indicates PAA MTs. Mto1-GFP itself is too faint to be seen. (D) Localization of small Mto1 C-terminal fragments fused to GFP plus tetrameric coiled coil from human VASP (VTD). Arrowheads in merged images indicate absence of GFP-VTD-Mto1 fragments. (E) Summary of localization of Mto1 GFP-insertion and GFP-fusion protein fragments. Blue boxes denote predicted coiled-coil regions; purple box denotes MASC. CM1 and Mto2-binding regions of Mto1 are required for association with γ-tubulin complex and microtubule nucleation [13]. Additional data are shown in Figure S2. Scale bars represent 10 μm.

To determine whether MASC is sufficient for Mto1 localization, we expressed MASC-containing Mto1 fragments fused to GFP in *mto1*Δ cells. Large fragments such as GFP-Mto1(919-1115) showed robust localization to iSPBs, mSPBs, and eMTOC sites, as well as to MTs, but smaller fragments showed poor or no localization to specific sites (Figures 2A and 2E; Figure S2A). This indicates that additional regions of Mto1 N-terminal to MASC are important for Mto1 localization. Further experiments in which GFP was inserted between nonlocalizing Mto1 N-terminal fragments and C-terminal fragments (Figures 2B, 2C, and 2E; Figures S2B–2D) revealed that different, nonoverlapping regions N-terminal to MASC can help in localization (Figure 2E, compare GFP-Mto1(1007-1115) to GFP-Mto1(919-1115) and Mto1(1-800)-GFP-Mto1(1028-1115)). These regions contain predicted coiled coils, leading us to hypothesize that coiled coil-dependent multimerization may be critical for MASC-dependent Mto1 localization, consistent with evidence that Mto1 interacts with itself and is present in large protein complexes in vivo (L. Groocock, A. Anders, and K.E.S., unpublished data). We fused three different heterologous coiled-coil sequences to the nonlocalizing GFP-Mto1(1007-1115) fragment: the dimeric C-terminal leucine zipper of budding yeast GCN4 (33 aa), which forms a conventional left-handed coiled-coil [19], and tetramerization domains from human vasodilator-stimulated phosphoprotein (VASP; 45 aa) [20] and *S. maritima* tetrabrachion (50 aa) [21], both of which form highly stable right-handed coiled coils. All three fusion proteins localized to SPBs and to eMTOC sites when expressed in *mto1*Δ cells (Figure S2E), demonstrating that sequence-independent multimerization promotes MASC-dependent Mto1 localization, most likely by increasing avidity of Mto1 binding to MTOC sites.

We used heterologous multimerization via the VASP tetramerization domain (VTD) to further define the minimal regions required for Mto1 localization to different sites. GFP-VTD-Mto1(1049-1075) localized to iSPBs but not mSPBs, whereas GFP-VTD-Mto1(1049-1095) localized to both iSPBs and mSPBs. GFP-VTD-Mto1(1028-1095) showed complete localization to iSPBs, mSPBs, and eMTOC sites (Figures 2D and 2E). These results indicate that a 68 aa region within the Mto1 C terminus, only slightly greater than MASC itself, contains multiple sequences regulating localization to three different prospective MTOC sites. The smallest of these, Mto1(1049-1075), comprises only 27 aa and supports localization to the iSPB.

### Mto1 eMTOC Localization Depends on Interaction with Unconventional Myosin Myp2

**Figure 3:**
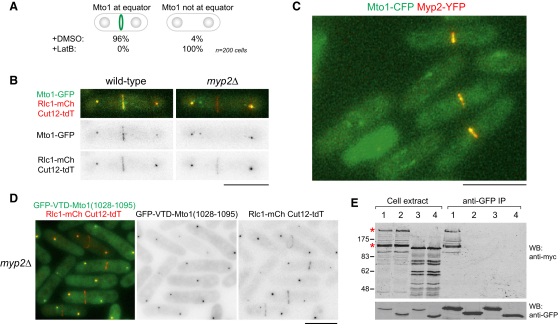
Mto1 Localization to eMTOCs Depends on Interaction with Type II Myosin Myp2 (A) Localization of GFP-Mto1(784-1115) to eMTOCs in binucleate cells treated with latrunculin B (LatB) or control (DMSO). (B) Mto1-GFP (green) colocalizes with the contractile actin ring (CAR) component Rlc1-mCherry (mCh, red) in dividing wild-type but not *myp2*Δ cells. SPB marker Cut12-tdT is also in red. (C) Colocalization of Mto1-CFP (green) with Myp2-YFP (red) in dividing cells. (D) Absence of GFP-VTD-Mto1(1028-1095) (green) from eMTOC sites in *myp2*Δ cells. Rlc1-mCh and Cut12-tdT are shown in red. (E) Coimmunoprecipitation of myc-tagged Myp2 with GFP-VTD-Mto1(1028-1095) by anti-GFP antibody. Western blots were probed with anti-myc (top) and anti-GFP (bottom). Lane 1: GFP-VTD-Mto1(1028-1095), Myp2-myc; lane 2: GFP-VTD-Mto1(1049-1095), Myp2-myc; lane 3: GFP-VTD-Mto1(1028-1095), Sid4-myc; lane 4: GFP-VTD-Mto1(1028-1065), Sid4-myc. Asterisks indicate full-length Myp2-myc and a C-terminal degradation product. Additional data are shown in Figure S3. Scale bars represent 10 μm.

We found that Mto1 localization to eMTOC sites was abolished by disruption of the actin cytoskeleton with latrunculin B (Figure 3A) and that Mto1 colocalized with markers of the contractile actin ring (CAR) during cytokinesis (Figure 3B; Figure S3A), suggesting that Mto1 localization to eMTOC sites depends on association with a CAR component. Although CAR assembly initiates relatively early during cell division [22], Mto1 localized to eMTOC sites only after anaphase onset (Figure 1G; additional data not shown). To identify potential CAR components involved in Mto1 eMTOC localization, we assayed Mto1-GFP localization in deletion mutants of proteins associating with the CAR during later stages of division [22]. Localization was normal in mutants of the septin Spn1 [23] and actin capping protein Acp2 [24]. However, in mutants of the type II myosin Myp2 [25, 26], Mto1-GFP eMTOC localization was abolished, whereas SPB localization was unaltered (Figure 3B; Figure S3B). Although GFP-tagged Alp4 was observed at eMTOC sites in ∼66% of wild-type cells with a CAR, it was completely absent from eMTOC sites in *myp2*Δ cells (data not shown; see Supplemental Experimental Procedures); accordingly, *myp2*Δ mutants failed to nucleate PAA MTs (Figure S3C). Localization of Myp2 to the CAR was not dependent on Mto1 (data not shown).

Colocalization of Mto1-CFP with Myp2-YFP at the CAR (Figure 3C) suggested that Mto1 and Myp2 may physically interact. We found that Mto1 coimmunoprecipitated with Myp2-YFP in fission yeast cell extracts (Figure S3E), and Myp2 interacted with the Mto1 C terminus in yeast two-hybrid assays in a MASC-dependent manner (Figure S3D). Localization of GFP-VTD-Mto1(1028-1095) to eMTOC sites (but not to SPBs) was also strictly dependent on Myp2 (Figure 3D), and GFP-VTD-Mto1(1028-1095) coimmunoprecipitated myc-tagged Myp2, whereas GFP-VTD-Mto1(1049-1095), which does not localize to eMTOCs, did not (Figure 3E).

An interaction involving Mto1 and Myp2 thus controls Mto1 eMTOC localization and formation of PAA MTs, providing a molecular explanation for earlier observations that PAA MT nucleation requires an intact CAR [27]. Because our results are based on coimmunoprecipitation and two-hybrid interaction, we cannot fully exclude the possibility that the Mto1-Myp2 interaction is mediated by a third protein, but this may be considered unlikely. Myp2 is an unusual type II myosin; unlike the essential fission yeast type II myosin Myo2, Myp2 plays no essential role in ring contraction and is required for viability only under stress conditions [25, 26]. Our results indicate a novel role for Myp2 in organizing the postanaphase MT cytoskeleton.

### Mto1 Localization to the Mitotic SPB Requires a Septation Initiation Network-Independent Function of Cdc11

**Figure 4:**
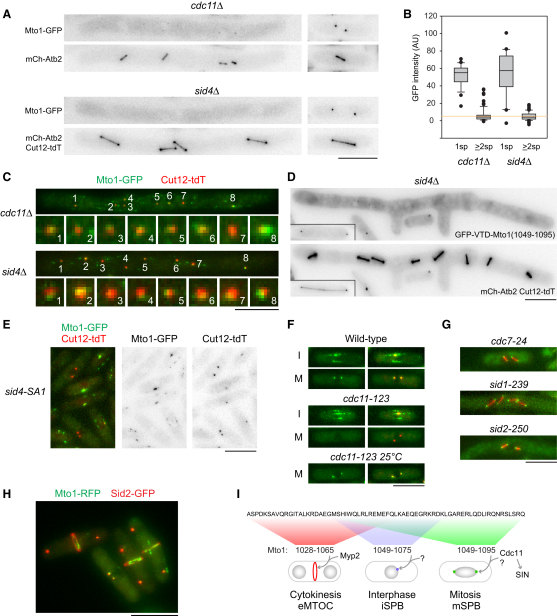
Sid4 and Cdc11 Regulate Mto1 mSPB Localization Independently of Their Role in the Septation Initiation Network (A) Absence or near absence of Mto1-GFP from mSPBs in multinucleate mitotic *cdc11*Δ and *sid4*Δ cells (left), with spindles and/or spindle poles shown underneath (mCh-Atb2, Cut12-tdT). Mononucleate cells from the same culture (e.g., cells that have not lost rescuing plasmids) retain Mto1-GFP at SPBs (right). (B) Quantitation of Mto1-GFP mSPB signal in early-to-mid mitotic cells from the experiment in (A), scoring mononucleate one-spindle (1sp) and multinucleate two-or-more-spindle (≥2sp) cells. Error bars show interdecile range. Orange line shows upper bound (95th percentile) from comparable measurements of non-SPB background areas. (C) Mto1-GFP (green) colocalizes with SPB marker Cut12-tdT (red) at iSPBs in *cdc11*Δ and *sid4*Δ. Enlarged images of each SPB are shown underneath. (D) GFP-VTD-Mto1(1049-1095) has strongly reduced mSPB localization in multinucleate mitotic *sid4*Δ cells. Insets show mononucleate mitotic cell from the same culture. (E) Mto1-GFP is present at iSPBs, but not at mSPBs, in *sid4-SA1* mutants at 36°C. (F) Mto1-GFP SPB localization (green) in interphase (I) and mitosis (M) in wild-type and *cdc11-123* mutants at 25°C and 36°C. Right column shows merge with SPB marker Sad1-dsRed (red). (G) Mto1-GFP (green) is present at mSPBs in *cdc7-24*, *sid1-239*, and *sid2-250* mutants at 36°C. RFP-Atb2 spindles are shown in red. (H) Mto1-RFP (green) and Sid2-GFP (red) localize to different equatorial structures during septation. (I) Model for generation of diversity of MTOC by multiple localization signals in the Mto1 C terminus and different cognate *trans*-acting factors. Additional data are shown in Figure S4. Scale bars represent 10 μm, except (C) insets, which represent 2 μm.

Sid4 and Cdc11 are SPB-associated scaffold proteins that recruit signaling proteins of the septation initiation network (SIN), which is involved in septum formation ([28, 29]; see below). Mto1-GFP was almost completely absent from mSPBs in *cdc11*Δ cells with a multinucleate SIN phenotype, and similar results were obtained with *sid4*Δ mutants, in which Cdc11 protein is present but not localized to SPBs (Figures 4A and 4B) [30, 31]. Low levels of Mto1 at some mSPBs in *cdc11*Δ cells may be due to residual levels of Cdc11 in some cells (Supplemental Experimental Procedures). Mto1-GFP localized normally to iSPBs in both *cdc11*Δ and *sid4*Δ mutants (Figure 4C; Supplemental Experimental Procedures), indicating that the requirement for Cdc11 and Sid4 is specific to mitosis. GFP-VTD-Mto1(1028-1095) and GFP-VTD-Mto1(1049-1095) also showed a strong reduction in mSPB localization in *cdc11*Δ and *sid4*Δ mutants, whereas their localization to iSPB and eMTOC sites was not affected (Figure 4D; Figures S4E and S4F).

Our finding that Cdc11 and Sid4 are required for Mto1 mSPB localization initially appeared to be inconsistent with the initial characterization of *cdc11*Δ mutants, in which astral MTs were described to be abnormal but nevertheless present [30]. However, further investigations suggest that apparent differences can be accounted for and that some experiments in the initial characterization of *cdc11*Δ may have been misinterpreted (Supplemental Experimental Procedures and Figures S4A–S4D).

Sid4 and Cdc11 are thought to serve primarily as a platform for “downstream” signaling components of SIN, including the small GTPase Spg1 and protein kinases Cdc7, Sid1, and Sid2, which transmit signals from SPBs to the septum at the cell division site [28, 29]. Interestingly, Mto1-GFP localized normally to mSPBs in *cdc7-ts*, *sid1-ts*, and *sid2-ts* mutants (Figures 4E–4G; Figure S4G). This bifurcation of phenotypes indicates that Sid4 and Cdc11 control multiple divergent pathways, one regulating SIN and the other regulating Mto1 mSPB localization. Consistent with this view, we found that even though Mto1 and Sid2 both localize to the cell-division site, they do not colocalize; Mto1 associates with the contracting CAR (via Myp2), whereas Sid2 associates with the developing septum more peripherally (Figure 4H).

Because Sid4 is required for Cdc11 SPB localization, the role of Sid4 in Mto1 mSPB localization may be indirect. In a yeast two-hybrid screen, we identified an interaction between the Mto1 C terminus and a C-terminal fragment of Cdc11, which contains 16–17 leucine-rich repeats (Figures S4H and S4I). Mto1 truncations interacting with Cdc11 correlated with those supporting mSPB localization, whereas truncations from either end of the Cdc11 fragment abrogated interaction (Figures S4H and S4I). Because the interaction between Mto1 and Cdc11 was relatively weak in semiquantitative assays (Figure S4H) and attempts to confirm it biochemically have not been successful (data not shown), its physiological significance remains unclear. In budding yeast, the MASC-containing protein Spc72p (Figure S1A; see above) interacts with Nud1p, an SPB outer plaque protein that contains leucine-rich repeats and resembles fission yeast Cdc11 [31, 32]. Because Nud1p is also involved in the SPB localization of budding homologs of SIN proteins (i.e., components of the mitotic exit network; [33]), there may be similarities between these two systems. One interesting, albeit speculative, possibility is that Cdc11 acts as a “coreceptor” that is necessary but not sufficient for Mto1 mSPB localization. Identification of additional proteins directing Mto1 SPB localization will be important in testing this hypothesis.

### Conclusions

Spatially and temporally regulated MT organization in eukaryotic cells depends largely on localizing MT nucleation to specific subcellular sites. Here we have shown how a modular system involving fission yeast Mto1 allows its association with diverse prospective MTOC sites to convert them into active MTOCs (Figure 4I). These principles may serve as a useful paradigm for understanding the generation of MTOC diversity in higher eukaryotes.

## Supplemental Information

Document S1. Supplemental Experimental Procedures and Four Figures

Document S2. Table S1

Movie S1. Related to Figure 1. GFP-Tubulin (GFP-Atb2) Dynamics in mto1(1-1051)-GFP Cells Wide-field images were taken every 15 s and deconvolved. Time compression is 150×.

Movie S2. Related to Figure 1. GFP-Tubulin (GFP-Atb2) Dynamics in mto1(1-1095)-GFP Cells Wide-field images were taken every 15 s and deconvolved. Time compression is 150×.
