# Supplementary material for: Fission Yeast Mto1 Regulates Diversity of Cytoplasmic Microtubule Organizing Centers
Source: Curr Biol. 2010 Nov 9;20(21):1959–65. doi: 10.1016/j.cub.2010.10.006 (PMC2989437; doi:10.1016/j.cub.2010.10.006)
Supplement: Document S1. Supplemental Experimental Procedures and Four Figures [file mmc1.pdf]

Current Biology, Volume *20*

Supplemental Information

Fission Yeast Mto1 Regulates Diversity of Cytoplasmic Microtubule Organizing Centers

Itaru Samejima, Victoria J. Miller, Sergio A. Rincon, and Kenneth E. Sawin

Figure S1

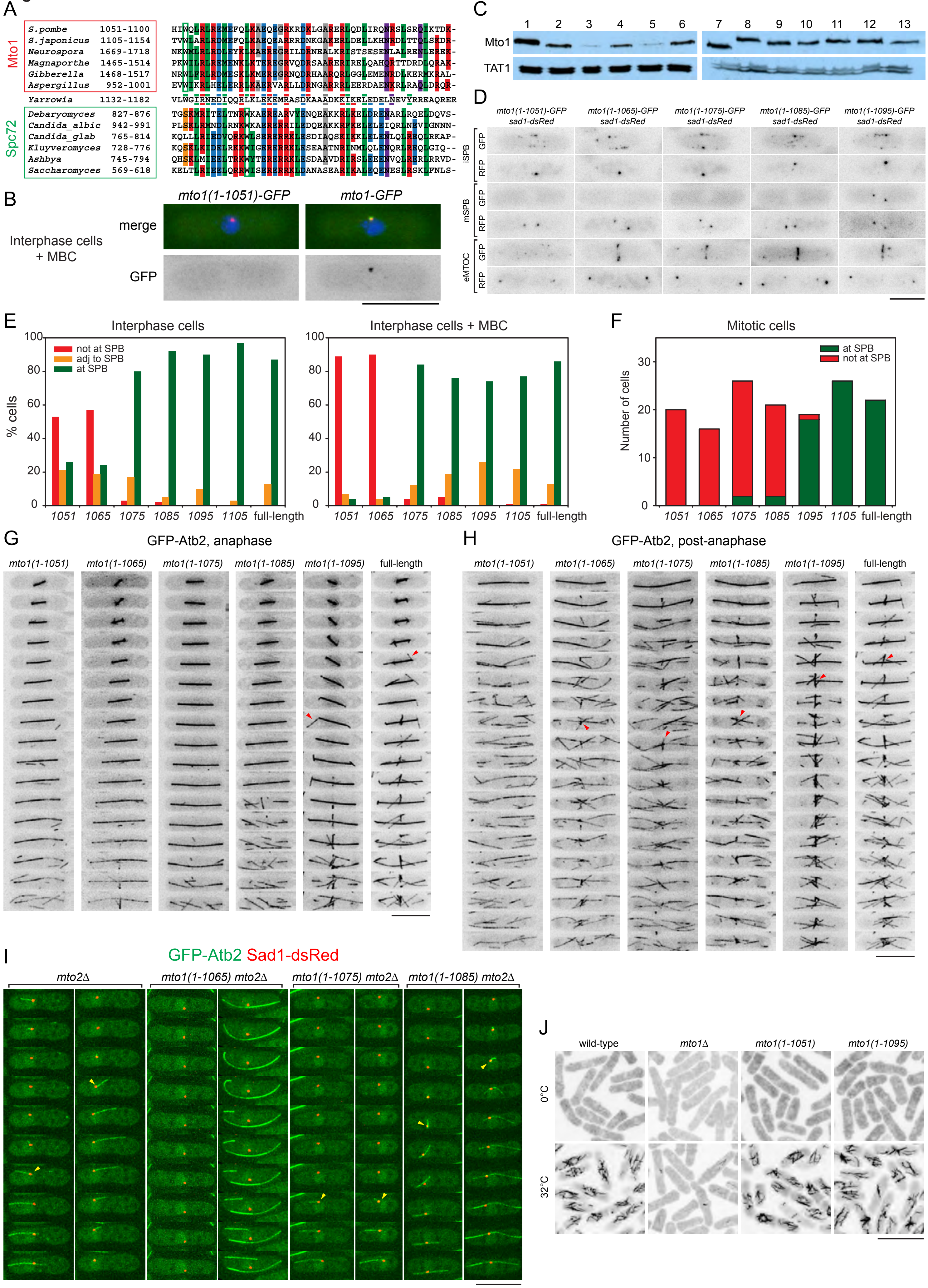
A
Mto1
S.pombe 1051-1100
S.japonicus 1105-1154
Neurospora 1669-1718
Magnaporthe 1465-1514
Gibberella 1468-1517
Aspergillus 952-1001
Yarrowia 1132-1182
Spc72
Debaryomyces 827-876
Candida_albic 942-991
Candida_glab 765-814
Kluyveromyces 728-776
Ashbya 745-794
Saccharomyces 569-618
B
mto1(1-1051)-GFP
mto1-GFP
Interphase cells + MBC
merge
GFP
C
1 2 3 4 5 6 7 8 9 10 11 12 13
Mto1
TAT1
D
mto1(1-1051)-GFP sad1-dsRed
mto1(1-1065)-GFP sad1-dsRed
mto1(1-1075)-GFP sad1-dsRed
mto1(1-1085)-GFP sad1-dsRed
mto1(1-1095)-GFP sad1-dsRed
iSPB
mSPB
eMTOC
GFP
RFP
E
Interphase cells
Interphase cells + MBC
% cells
not at SPB
adj to SPB
at SPB
1051 1065 1075 1085 1095 1105 full-length
F
Mitotic cells
Number of cells
at SPB
not at SPB
1051 1065 1075 1085 1095 1105 full-length
G
GFP-Atb2, anaphase
mto1(1-1051) mto1(1-1065) mto1(1-1075) mto1(1-1085) mto1(1-1095) full-length
H
GFP-Atb2, post-anaphase
mto1(1-1051) mto1(1-1065) mto1(1-1075) mto1(1-1085) mto1(1-1095) full-length
I
GFP-Atb2 Sad1-dsRed
mto2Δ
mto1(1-1065) mto2Δ
mto1(1-1075) mto2Δ
mto1(1-1085) mto2Δ
J
wild-type
mto1Δ
mto1(1-1051)
mto1(1-1095)
0°C
32°C

Figure S2

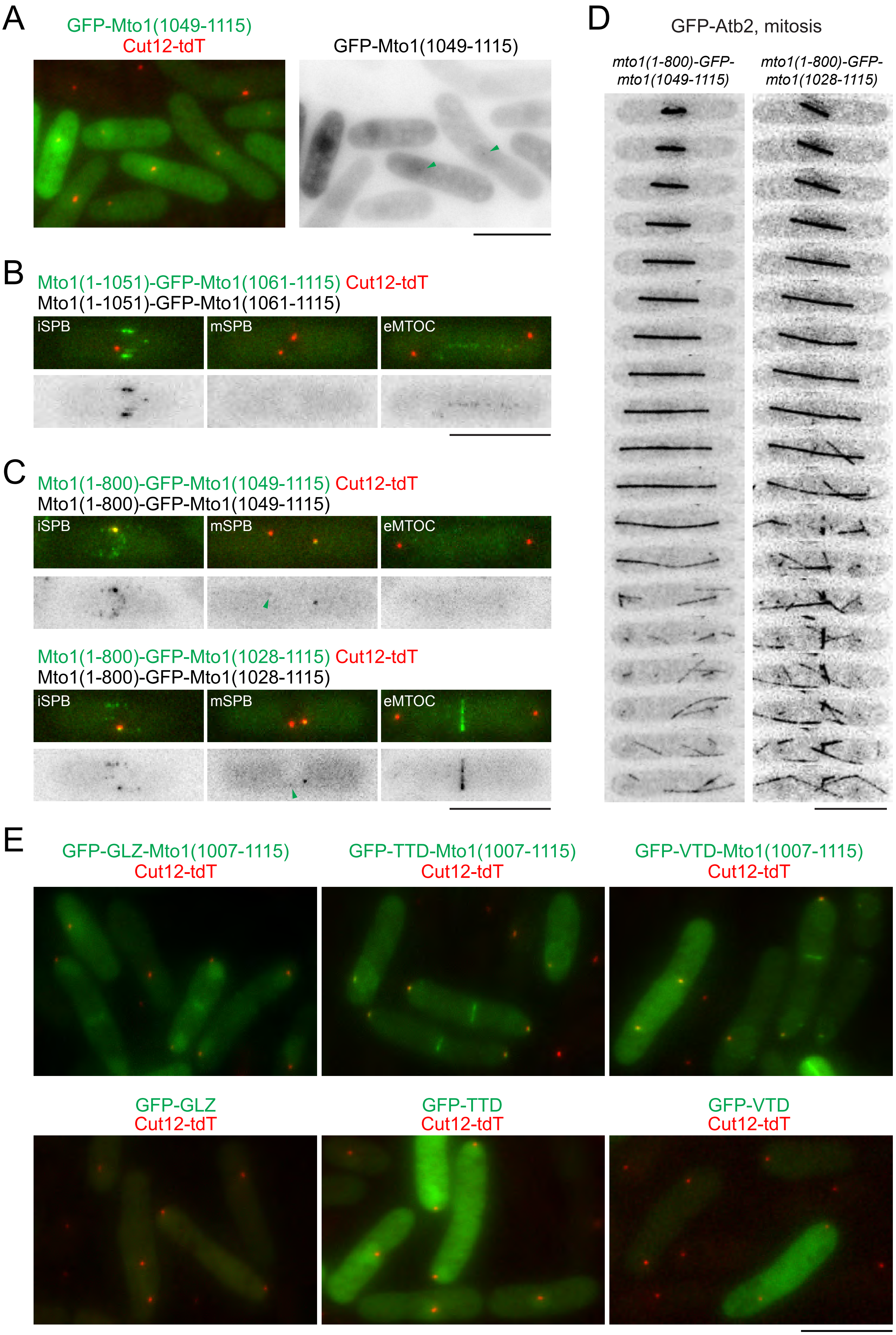
A
GFP-Mto1(1049-1115)
Cut12-tdT
GFP-Mto1(1049-1115)
B
Mto1(1-1051)-GFP-Mto1(1061-1115) Cut12-tdT
Mto1(1-1051)-GFP-Mto1(1061-1115)
iSPB
mSPB
eMTOC
C
Mto1(1-800)-GFP-Mto1(1049-1115) Cut12-tdT
Mto1(1-800)-GFP-Mto1(1049-1115)
iSPB
mSPB
eMTOC
Mto1(1-800)-GFP-Mto1(1028-1115) Cut12-tdT
Mto1(1-800)-GFP-Mto1(1028-1115)
iSPB
mSPB
eMTOC
D
GFP-Atb2, mitosis
mto1(1-800)-GFP-mto1(1049-1115)
mto1(1-800)-GFP-mto1(1028-1115)
E
GFP-GLZ-Mto1(1007-1115)
Cut12-tdT
GFP-TTD-Mto1(1007-1115)
Cut12-tdT
GFP-VTD-Mto1(1007-1115)
Cut12-tdT
GFP-GLZ
Cut12-tdT
GFP-TTD
Cut12-tdT
GFP-VTD
Cut12-tdT

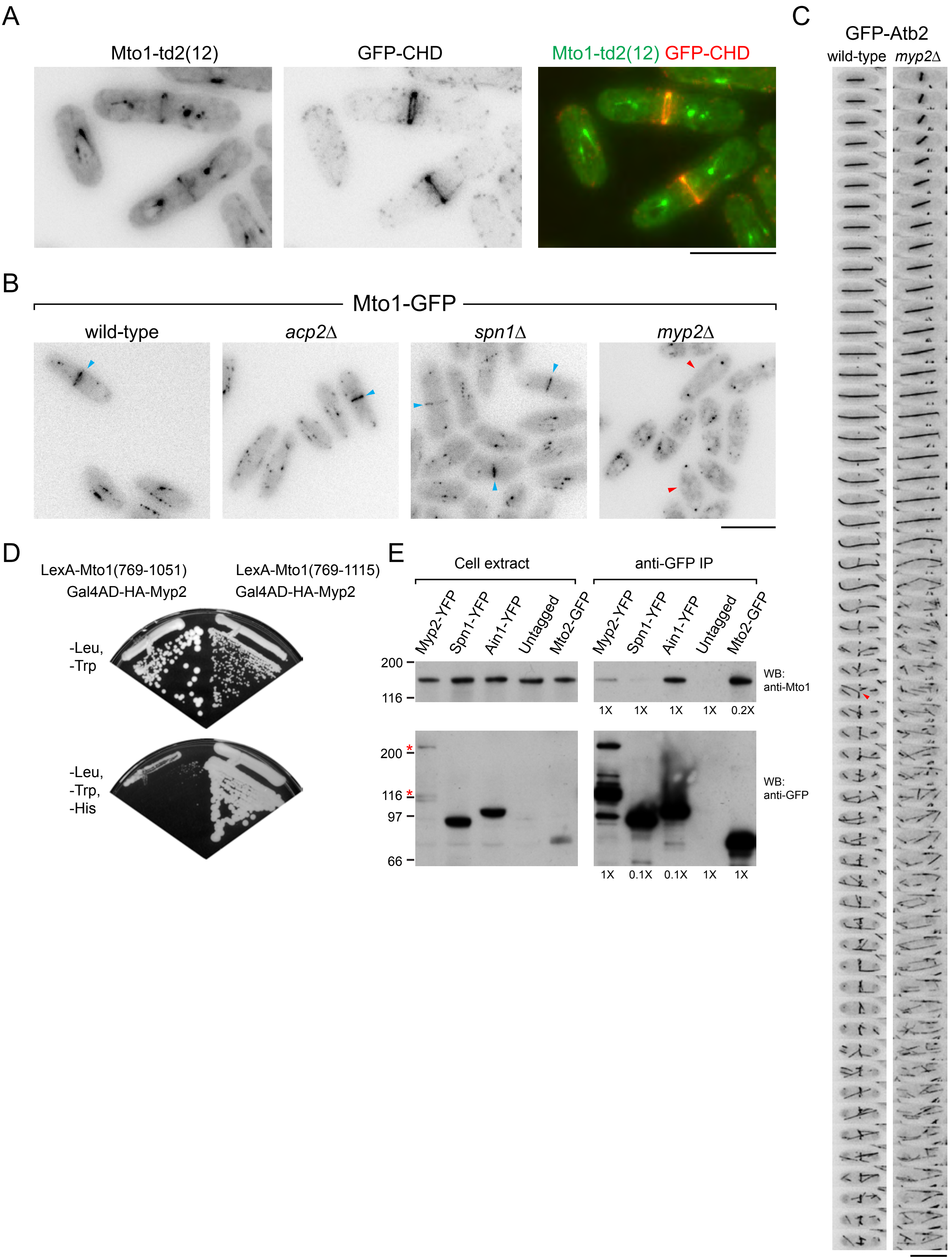
A
Mto1-td2(12)
GFP-CHD
Mto1-td2(12) GFP-CHD
B
Mto1-GFP
wild-type
acp2Δ
spn1Δ
myp2Δ
C
GFP-Atb2
wild-type
myp2Δ
D
LexA-Mto1(769-1051)
Gal4AD-HA-Myp2
LexA-Mto1(769-1115)
Gal4AD-HA-Myp2
-Leu,
-Trp
-Leu,
-Trp,
-His
E
Cell extract
anti-GFP IP
Myp2-YFP
Spn1-YFP
Ain1-YFP
Untagged
Mto2-GFP
200
116
WB:
anti-Mto1
1X 1X 1X 1X 0.2X
200 *
116 *
97
66
WB:
anti-GFP
1X 0.1X 0.1X 1X 1X

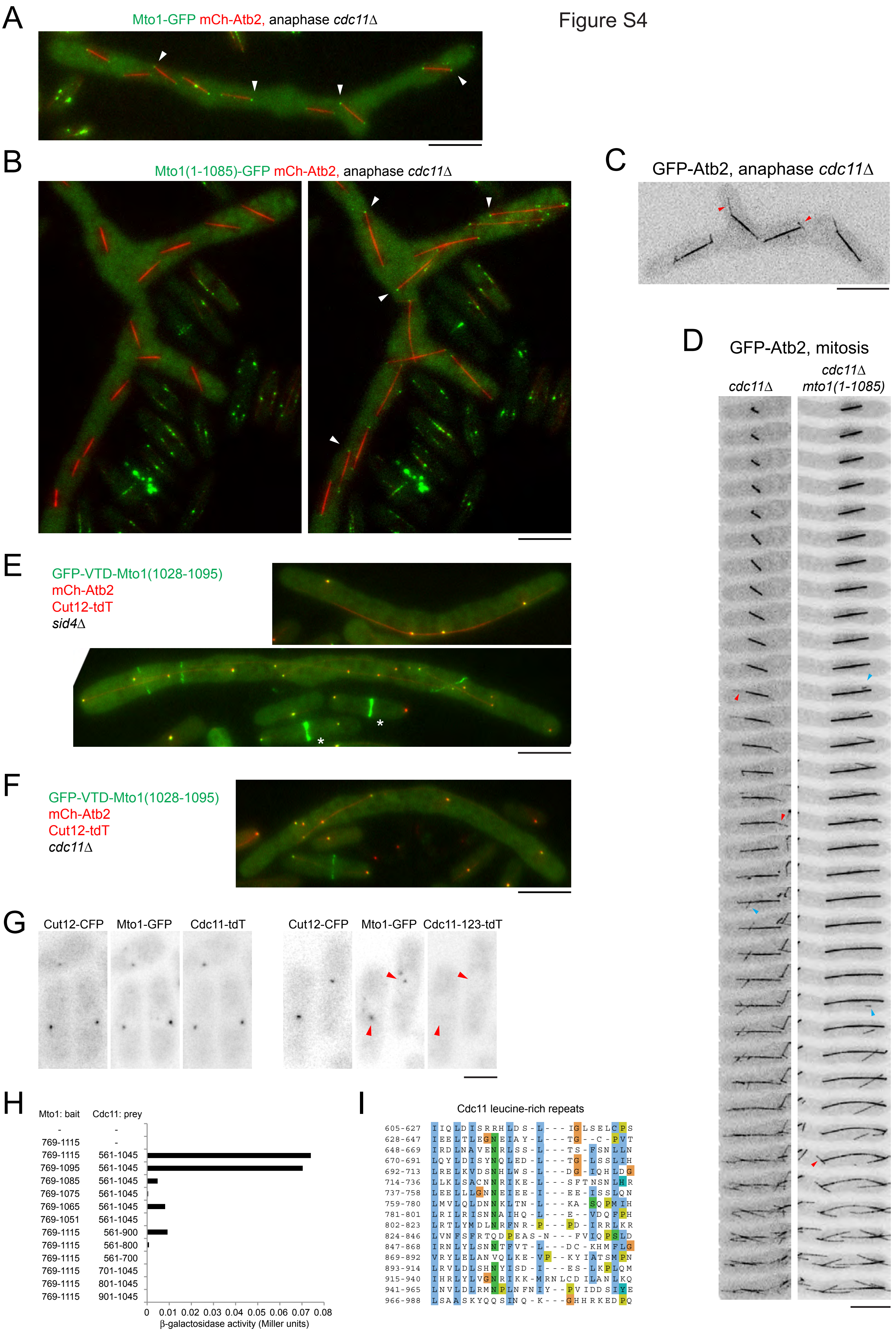
A
Mto1-GFP mCh-Atb2, anaphase cdc11Δ
B
Mto1(1-1085)-GFP mCh-Atb2, anaphase cdc11Δ
C
GFP-Atb2, anaphase cdc11Δ
D
GFP-Atb2, mitosis
cdc11Δ
cdc11Δ mto1(1-1085)
E
GFP-VTD-Mto1(1028-1095)
mCh-Atb2
Cut12-tdT
sid4Δ
*
*
F
GFP-VTD-Mto1(1028-1095)
mCh-Atb2
Cut12-tdT
cdc11Δ
G
Cut12-CFP
Mto1-GFP
Cdc11-tdT
Cut12-CFP
Mto1-GFP
Cdc11-123-tdT
H
Mto1: bait
Cdc11: prey
- -
769-1115 -
769-1115 561-1045
769-1095 561-1045
769-1085 561-1045
769-1075 561-1045
769-1065 561-1045
769-1051 561-1045
769-1115 561-900
769-1115 561-800
769-1115 561-700
769-1115 701-1045
769-1115 801-1045
769-1115 901-1045
0 0.01 0.02 0.03 0.04 0.05 0.06 0.07 0.08
β-galactosidase activity (Miller units)
I
Cdc11 leucine-rich repeats
605-627 IIQLDISRRHLDS-L---IGLSELCPS
628-647 IEELTLEGNEIAY-L---TG--C-PVT
648-669 IRDLNAVENRLSS-L---TS-FSNLLN
670-691 LQYLDISYNQLED-L---TG-LSSLIH
692-713 LRELKVDSNHLWS-L---DG-IQHLDG
714-736 LLKLSACNNRIKE-L---SFTNSNLHR
737-758 LEELLLGNNEIEE-I---EE-ISSLQN
759-780 LMVLQLDNNKLTN-L---KA-SQPMIH
781-801 LRILRISNNAIHQ-L---E--VDQFPH
802-823 LRTLYMDLNRFNR-P---PD-IRRLKR
824-846 LVNFSFRTQDPEAS-N---FVIQPSLD
847-868 IRNLYLSNNTFVT-L---DC-KHMFLG
869-892 VRYLELANVQLKE-VP--KYIATSMPN
893-914 LRVLDLSHNYISD-I---ES-LKPLQM
915-940 IHRLYLVGNRIKK-MRNLCDILANLKQ
941-965 LNVLDLRMNPLNFNIY--PVIDDSIYE
966-988 LSAASKYQQSINQ-K---GHHRKEDPQ

Supplemental Figure Legends

Figure S1, Related to Figure 1.

(A) Evolutionary conservation of Mto1 C-terminus. Alignment of amino acid sequences divides MASC-containing proteins into three families (Mto1, Spc72, Yarrowia). Similar amino acids within families are colored. Residues conserved between Yarrowia and other families are indicated by colored bars above and below the Yarrowia sequence.

(B) Absence of Mto1(1-1051)-GFP from interphase SPB in MBC-treated cells. Merged images shows Mto1(1-1051)-GFP and Mto1-GFP (green) together with SPB marker Sad1-dsRed (red), and DAPI staining (blue), after MBC treatment; see also Fig. S1E.

(C) GFP-tag at the C-terminus stabilizes Mto1-truncation mutants. Anti-Mto1 western blots of untagged and GFP-tagged Mto1-truncation mutants. Lanes 1 and 7, full-length wild-type Mto1; lane 2, Mto1(1-1051); lane 3, Mto1(1-1065); lane 4, Mto1(1-1075); lane 5, Mto1(1-1085); lane 6, Mto1(1-1095); lane 8, Mto1-GFP; lane 9, Mto1(1-1051)-GFP; lane 10, Mto1(1-1065)-GFP; lane 11, Mto1(1-1075)-GFP; lane 12, Mto1(1-1085)-GFP; lane 13, Mto1(1-1095)-GFP. Anti-tubulin (TAT1) western blot serves as a loading control.

(D) Single-channel images of localization of Mto1-GFP truncation mutants. For clarity, the images shown in Fig. 1E are reproduced here as single-channel images, showing presence or absence of truncations at interphase SPBs (iSPB), mitotic SPBs (mSPB) and equatorial MTOCs (eMTOC). SPB marker is Sad1-dsRed.

(E) Interphase SPB localization of Mto1-GFP truncation mutants. Cells also expressed the SPB marker Sad1-dsRed. To rule out false-positive SPB localization due to association of Mto1 with MTs near SPBs, experiments were done both in the absence (left) and presence (right) of the microtubule-depolymerizing drug MBC.
Shown are: Mto1(1-1051)-GFP; Mto1(1-1065)-GFP; Mto1(1-1075)-GFP; Mto1(1-1085)-GFP; Mto1(1-1095)-GFP; Mto1(1-1105)-GFP; full-length Mto1-GFP.

(F) Mitotic SPB localization of Mto1-GFP truncation mutants. Cells also expressed mCherry-tubulin (Atb2) to identify mitotic spindles. Shown are: Mto1(1-1051)-GFP; Mto1(1-1065)-GFP; Mto1(1-1075)-GFP; Mto1(1-1085)-GFP; Mto1(1-1095)-GFP; Mto1(1-1105)-GFP; full-length Mto1-GFP.

(G,H) Cytoplasmic microtubule nucleation in anaphase and post-anaphase in wild type and *mto1*-truncation mutants. Time-lapse images (1-min intervals) of GFP-tubulin in *mto1(1-1051)-GFP*; *mto1(1-1065)-GFP*; *mto1(1-1075)-GFP*; *mto1(1-1085)-GFP*; *mto1(1-1095)-GFP*; full length *mto1-GFP*. Arrowheads show astral MTs (F) and PAA MTs (G).

(I) *mto1(1-1065)-GFP* mutants do not nucleate MTs from the interphase SPB. Time-lapse images (20-sec intervals) of GFP-tubulin (green) and SPB marker Sad1-dsRed (red) in *mto2*Δ, *mto1(1-1065)-GFP mto2*Δ, *mto1(1-1075)-GFP mto2*Δ, and *mto1(1-1085)-GFP mto2*Δ mutants. *mto2*Δ mutant backgrounds were used because *mto2*Δ mutants are defective in non-SPB MT nucleation and thus can nucleate interphase MTs *only* from the SPB [9]. GFP signal from Mto1 itself is too faint to be seen here. Arrowheads show interphase microtubule nucleation from the SPB.

(J) Mto1 C-terminus is not required for microtubule nucleation *per se*. MT-regrowth experiment showing anti-tubulin immunofluorescence after 30 minutes cold-treatment (0°C, top row) and then one minute after temperature shift-up (32°C, bottom row), for wild-type, *mto1*Δ, *mto1(1-1051)* and *mto1(1-1095)* cells. MTs return to steady-state distributions within about 10 minutes in wild-type, *mto1(1-1051)* and *mto1(1-1095)* cells [7].
All bars, 10 μm.

Figure S2, Related to Figure 2.
(A) Localization of GFP- Mto1(1049-1115). In some cells a weak signal is detected at interphase SPBs (arrowheads). SPB marker Cut12-tdT is shown in red.
(B) "GFP-insertion" Mto1(1-1051)-GFP-Mto1(1061-1115), lacking the N-terminal portion of MASC, does not localize to iSPBs, mSPBs or eMTOC. SPB marker Sad1-dsRed is shown in red.
(C) Localization of "GFP-insertion" proteins Mto1(1-800)-GFP-Mto1(1049-1115) (top) and Mto1(1-800)-GFP-Mto1(1028-1115) (bottom). Both localize strongly to iSPBs but often weakly to one or both of the two mSPBs (arrowheads). Only Mto1(1-800)-GFP-Mto1(1028-1115) localizes to eMTOC. SPB marker Cut12-tdT is shown in red.
(D) Time-lapse images (1-min intervals) of GFP-tubulin in the Mto1 GFP-insertion mutants shown in (C). Astral MTs are only occasionally observed. PAA MTs are observed in *mto1(1-800)-GFP-mto1(1028-1115).*
(E) Coiled-coil sequences significantly enhance localisation of Mto1 C-terminal fragments to SPBs and eMTOC. Localization of fusion proteins containing coiled-coil sequences from GCN4 leucine zipper domain (GLZ), tetrabrachion (TTD), or VASP tetramerization domain (VTD) inserted between GFP and Mto1(1007-1115) (top). Compare with Fig. 2A. Also shown are coiled-coil sequences plus GFP alone (bottom). SPB marker Cut12-tdT is shown in red.
All bars, 10 μm.

Figure S3, Related to Figure 3.
(A) Colocalization of Mto1-GFP with contractile actin ring (CAR) during cell division. Mto1-td2(12) (RFP; [34]) co-imaged with a GFP-fusion to the calponin-homology domain (CHD) of IQGAP protein Rng2, a marker for actin cables [35].
(B) Absence of Mto1-GFP from eMTOC sites in *myp2*Δ. Mto1-GFP eMTOC localization assayed in deletion mutants of proteins that associate with the CAR at the same time as Mto1 during cell division. Arrowheads indicate presence (blue) and absence (red) of Mto1-GFP from eMTOC sites.
(C) Time-lapse images (30-sec intervals) of GFP-tubulin during wild-type and *myp2*Δ cell division. PAA MTs (arrowhead, left) were observed in 14/14 wild-type cell divisions but in 0/12 *myp2*Δ cell divisions.
(D) Yeast two-hybrid interaction of Mto1 C-terminal fragments with Myp2. Fragment Mto1(769-1115) interacts with Myp2 (growth on –Leu, -Trp, -His; right bottom), but fragment Mto1(769-1051), which lacks MASC, does not (left bottom).
(E) Mto1 is co-immunoprecipitated with Myp2-YFP. Anti-GFP immunoprecipitations from indicated strains, probed with antibodies to Mto1 and GFP. "Cell extract" lanes (left) are loaded with equal amounts of cell extract. To compensate for different endogenous expression levels of YFP- and GFP-tagged proteins, "anti-GFP IP" lanes (right) are loaded with different amounts of cell-extract equivalents. "1X" indicates 100-fold loading relative to cell extracts; "0.1X" indicates 10-fold loading relative to cell extracts, etc. Ain1 (alpha-actinin) was independently found to interact with Mto1 in a yeast two-hybrid screen (not shown). Asterisks indicate full-length Myp2-YFP and major degradation products. Molecular weights are shown in kDa.
All bars, 10 μm.

Figure S4, Related to Figure 4.
(A) Mto1-GFP returns to SPBs in late-anaphase spindles in *cdc11*Δ.
Multinucleate cell. Arrowheads mark representative SPBs. Mto1-GFP (green), mCherry-tubulin (red).
(B) Mto1(1-1085)-GFP accumulates at SPBs in late-anaphase spindles in *cdc11*Δ. Two images of same multinucleate cell, 5 min apart. Arrowheads mark representative SPBs. Mto1(1-1085)-GFP (green), mCherry-tubulin (red).
(C) Astral MTs in late-anaphase spindles in *cdc11*Δ. Multinucleate cell. Arrowheads mark representative astral MTs.
(D) Astral and "pseudo-astral" MTs in *cdc11*Δ. Time-lapse images of GFP-tubulin from mitotic *cdc11*Δ and *cdc11*Δ *mto1(1-1085)-GFP* cells (30-sec intervals). Images are sub-regions of larger multi-nucleate cells; the *cdc11*Δ spindle shown is the rightmost part of the multinucleate cell in panel C. In addition to astral MTs nucleated from SPBs (red arrowheads), other MTs that could be mistaken for astral MTs are nucleated from non-SPB sites (blue arrowheads).
(E) GFP-VTD-Mto1(1028-1095) localisation in *sid4*Δ. The Mto1-fragment (green) localizes to SPBs in a multinucleate interphase cell (top) and to SPBs and eMTOC sites in a multinucleate late-mitotic cell (bottom). mCherry-tubulin and SPB marker Cut12-tdT are shown in red. Localization to eMTOC sites is weak compared to mononucleate cells that have not yet lost rescuing plasmids (asterisks), most likely because of CAR instability in SIN mutants [36].
(F) GFP-VTD-Mto1(1028-1095) localization in *cdc11*Δ. The Mto1-fragment (green) localizes to SPBs in a multinucleate interphase cell. mCherry-tubulin and Cut12-tdT are shown in red.
(G) Mto1-GFP remains at iSPBs in *cdc11-123* mutants. Triple-label images of Cut12-CFP, Mto1-GFP, and either wild-type Cdc11-tdT (left) or mutant Cdc11-123-tdT (right), at restrictive temperature (36°C). Note absence of Cdc11-123-tdT from SPBs (arrowheads). Cells were treated with MBC to disrupt microtubules, ensuring validity of Mto1 localization.
(H) Yeast-two hybrid interaction of Mto1 C-terminal fragments with Cdc11(561-1045). Semi-quantitative assays using ortho-nitrophenyl beta-galactoside (ONPG) as substrate. Mto1 fragments that interact with Cdc11 correspond to those that localize to mSPBs (Fig. 1). Truncation of the Cdc11 fragment from either end abrogates the interaction.
(I) Position of leucine-rich repeats in Cdc11 C-terminus. While the Cdc11 C-terminus has been noted to contain leucine-rich repeats [31], our analysis suggests that these repeats are more extensive and continuous than previously recognized. Relevant amino acids are color-coded by similarity. Leucine-rich repeats typically fold into a single curved solenoid structure [37, 38], which may explain why truncation from either end of the Cdc11 fragment abrogates interaction with Mto1.
All bars, 10 μm.

Table S1. A list of fission yeast strains used in this work.
(See accompanying Excel spreadsheet.)

Supplemental Experimental Procedures

Yeast Strains

Standard fission yeast classical and molecular-genetic techniques were used throughout [39, 40]. **Table S1** contains a list of strains used. Deletion, truncation and tagging of genes at N- and C-termini were performed using PCR-based targeting methods [41]. Fluorescent tags included GFP as well as dsRed, mCherry (mCh), tandem dimer Tomato (tdT), and tandem dimer 2(12) (td2(12))[34, 42, 43]. Additional strains were purchased from Bioneer or derived from strains kindly provided by P. Fantes (University of Edinburgh, UK) T. Pollard (Yale University, USA), K. Gould (Vanderbilt University, USA), and M. Balasubramanian and S. Oliferenko (Temasek Life Science Laboratory, Singapore). Strains were confirmed by PCR and Western blotting as appropriate.

To make the triple-point mutant *mto1-427*, a 1.4 kb fragment covering the *mto1+* C-terminal region and containing mutation sites R1056A E1059A E1061A was generated by PCR. The PCR product was then used to transform an *mto1(1-1051):ura4+* strain (KS2696) to replace the *ura4+* cassette, selecting for resistance to 5-Fluoroorotic Acid (5-FOA). To make "GFP-insertion" strains, GFP-Mto1C-terminal fusion genes including *mto1* 3' untranslated sequence were amplified by PCR, and the PCR products were used to transform *mto1(1-1051)-GFP:ura4+* or *mto1(1-800)-GFP:ura4+* strains to replace the ura4 cassette, selecting for 5-FOA resistance.

To construct plasmids expressing GFP fused to Mto1-C-terminal fragments under control of the *nmt81* promoter [44], specified regions of Mto1 were amplified by PCR and subcloned 3' to the GFP-coding sequence of plasmid pKS71, an enhanced-GFP version of pWGA [45]. *mto1*Δ strains were used for plasmid transformation. To construct plasmids containing coiled-coil sequences between GFP and Mto1 sequences, synthetic DNA fragments (GeneArt) encoding coiled-coils were inserted at a SacII site in the linker sequence between GFP and Mto1 coding sequence in the above plasmids. Nucleotide sequences for the coiled-coil sequences are (SacII sites underlined):

(GCN4 leucine zipper):
5'CCGCGGCTGGTTCTGGTTCTGGATCTCGTATGAAACAACTTGAAGATAAGGTTG
AAGAACTTTTGTCTAAGAACTACCATCTTGAAAATGAAGTTGCTCGTCTTAAAAA
ACTTGTTGGTGAACGTGGATCTGGTTCAGGATCTGGTTCCGCGG3'

(Tetrabrachion tetramerization domain):
5'CCGCGGCTGGTTCTGGTTCTGGATCTATTATTAACGAAACTGCTGATGATATTG
TTTATCGTCTTACTGTTATTATTGATGATCGTTACGAATCTCTTAAAAATCTTATT
ACTCTTCGTGCTGATCGTTTAGAAATGATTATTAACGATAATGTTTCTACTATTCT
TGCTTCTATTGGATCTGGATCTGGTTCAGGTTCCGCGG3'

(VASP tetramerization domain):
5'CCGCGGCTGGTTCTGGTTCTGGATCTCCTTCTTCTTCTGATTATTCTGATCTTCA
ACGTGTTAAACAAGAATTGCTTGAAGAAGTTAAGAAAGAACTTCAAAAGGTTAA
AGAAGAAATTATTGAAGCTTTTGTTCAAGAATTACGTAAACGTGGTTCTCCTGGA
TCTGGATCTGGTTCAGGTTCCGCGG3'

To construct strains with GFP-VTD-Mto1-C-terminal fragments integrated at the *mto1* locus (in place of the endogenous *mto1+* gene), recombinant genes encoding various GFP-coiled-coil-Mto1-C-terminal fragments were amplified by PCR, using the multicopy plasmids described above as templates. Reverse PCR primers contained 80 nucleotides homology to a region 3' to *mto1+*. PCR products were used to transform an *nmt81:GFP-mto1(1-800):ura4+* strain, followed by 5-FOA selection. Homologous recombination within the GFP coding sequences and within *mto1+* 3' sequence replaced the *mto1(1-800):ura4+* sequence with coiled-coil-Mto1-C-terminal fragments (confirmed by sequencing).

Phenotypes of *cdc11*Δ and *sid4*Δ were analyzed primarily by loss of rescuing plasmids from haploid deletion strains, although in a few instances spore germination of heterozygous deletion strains was used. Heterozygous deletion strains *cdc11*Δ / *cdc11+* and *sid4*Δ / *sid4+* were created by replacing one copy of the respective ORFs with a *ura4+* cassette in diploid backgrounds. To generate rescuing plasmids, *cdc11+* and *sid4+* genes were amplified by genomic PCR and cloned into the *S. pombe* shuttle vector plasmid pAL-KS (selection for leucine prototrophy; [46, 47]). The plasmid pKS560 contains *cdc11+* coding sequence and 1091 bp of 5' sequence; pKS1031 contains *sid4+* coding sequence and 5' and 3' flanking sequences of 1065 bp and 165 bp, respectively. The heterozygous deletion strains were transformed with the appropriate plasmids and sporulated to generate haploid deletion strains containing the rescuing plasmid. For plasmid loss, haploid deletion strains containing the rescuing plasmid were cultured in medium supplemented with leucine, to allow growth of auxotrophs. For spore germination experiments (Suppl. Fig. 4C, 4D) heterozygous diploid cells (without rescuing plasmids) were sporulated on SPA plates. Asci were scraped from plates and treated with 0.2 % helicase. Spores were inoculated in YE5S and incubated at 32°C for 17.5 hrs before imaging.

Imaging and Physiology Experiments

For live-cell microscopy, EMM2 minimal medium with sodium glutamate as nitrogen source was used. Single time-point and time-lapse imaging of GFP- and RFP-fusion proteins was essentially as described previously [48, 49]. Cells were mounted on medium-agarose pads and sealed with VALAP before time-lapse imaging. Images were collected on a Nikon TE300 inverted microscope with automated filter and *z*-axis control, running MetaMorph software (Universal Imaging, Downingtown, PA). Time-lapse images were taken with intervals of 15-30 sec for single-channel and 20-30 sec for two-channel movies. Image sequences were further deconvolved using Softworx (Applied Precision, Issaquah, WA). Temperature-sensitive strains were observed using a heated objective at 36°C (Bioptechs).

For quantitation of Mto1-GFP and truncations at interphase SPBs (**Fig. S1E**), Sad1-dsRed was used as SPB marker. In some experiments (as indicated), to discriminate Mto1-GFP signal at the SPB from Mto1-GFP signal on microtubules near the SPB, microtubules were disrupted by addition of methyl benzimidazol-2-yl carbamate (MBC; 50 μg/ml final concentration), 30 min prior to imaging. 90-113 cells were scored for each strain. For quantitation of Mto1-GFP and truncations at mitotic SPBs (**Fig. S1F**), mCherry-tubulin (Atb2) was used both to confirm mitotic state and indicate spindle poles. In experiments with other Mto1-variants (**Fig. 2, Fig. S2**), additional quantitation of SPB localization was done using either Sad1-dsRed or Cut12-tdT as SPB marker (shown in table below):

To score Mto1-GFP and Mto1-427-GFP presence at eMTOC sites as a function of spindle length (**Fig. 1G** and additional data not shown), image projections were used to measure the

distance between SPBs (marked with Cut12-tdT) in mitotic cells, using MetaMorph software. 72 *mto1-GFP* and 61 *mto1-427-GFP* cells were scored. Based on the observed difference in Mto1-GFP localization in wild-type cells with short vs. long spindles (**Fig. 1G**), we used an SPB-SPB distance of > 8 μm as a criterion for additional quantitation of eMTOC localization of several Mto1-variants (shown in table below).

Additional quantitation for Mto1-variant localization (see ***Fig. 1, Fig. 2, Fig. S2)***

Mto1-variant	*at eMTOC**	*at iSPB*	*at mSPB*
Mto1-GFP	21/21 cells	(Fig. S1E)	(Fig. S1F)
Mto1(1-1065)-GFP	30/31	(Fig. S1E)	(Fig. S1F)
Mto1(1-1051)-GFP	0/28	(Fig. S1E)	(Fig. S1F)
Mto1(1-1051)-GFP-Mto1(1028-1115)	22/22	N/D	30/30 cells
Mto1(1-1051)-GFP-Mto1(1049-1115)	0/63	21/21 cells**	25/25
Mto1(1-1051)-GFP-Mto1(1061-1115)	0/16	5/24**	0/8
Mto1(1-800)-GFP-Mto1(1028-1115)	36/40	34/34**	18/25#
Mto1(1-800)-GFP-Mto1(1049-1115)	N/D	39/41**	8/11##
GFP-VTD-Mto1(1028-1095) †	16/23†	66/66	28/28
GFP-VTD-Mto1(1049-1095) †	0/14†	38/38	12/14
GFP-VTD-Mto1(1049-1075) †	0/4†	38/40	1/6††

* Mitotic cells with SPB-SPB distance > 8 μm were scored.
** Cells assayed without MBC treatment; see Fig. S1E and legend.
# In 10 of these 18 cells, Mto1 was at only one SPB.
In 4 of these 8 cells, Mto1 was at only one SPB.
† These Mto1-variants lack Mto1 N-terminus and therefore cells have aberrant cytoplasmic interphase microtubules and displaced nuclei, which indirectly affects mitosis and CARs. Therefore for eMTOC localization, mitotic cells with SPB-SPB distance > 5 μm were scored.
†† The single example at mSPB was a very late mitotic (early interphase?) cell in which only one SPB contained detectable signal.

To measure Mto1 at eMTOC sites after disruption of the actin cytoskeleton (**Fig. 3A**), cells expressed histone H3.2 (Hht2)-GFP to mark nuclei and mildly overexpressed *nmt81:*GFP-Mto1(784-1115) at the *mto1* locus in place of *mto1+*. Overexpression was necessary because of the relatively bright Hht2-GFP signal used to identify binucleate cells. Cells were treated with 100 μM Latrunculin B or with DMSO (carrier) for 30 min at 25°C prior to imaging. For each condition, 200 binucleate cells were scored for presence of absence of Mto1 at eMTOC sites.

Determination of Myp2-dependent Mto1 localization at the contractile actin ring (CAR; **Fig. 3B, 3D**) used an mCherry-tagged version of the CAR marker Rlc1 [50]. In wild-type cells, Mto1-GFP and GFP-VTD-Mto1(1028-1095) co-localized with Rlc1-mCh at the CAR in 7/9 cells and 11/11 cells, respectively, whereas in *myp2*Δ cells, both Mto1-GFP and GFP-VTD-Mto1(1028-1095) were absent from CARs containing Rlc1-mCh (0/3 cells and 0/31 cells, respectively). Mto1-CFP co-localized at the CAR with Myp2-YFP (**Fig. 3C**) in 30/34 cells.

Determination of Alp4 localization at the CAR in Mto1-truncation strains used tandem-dimer Tomato-tagged Alp4 [13]. In *mto1(1-1065)-GFP* cells, Alp4-tdT co-localized with Mto1(1-1065)-GFP at eMTOC sites in 15/15 late anaphase cells. By contrast, in *mto1(1-1051)-GFP* cells, Alp4-tdT was absent from eMTOC sites in 20/20 late anaphase cells. Alp4 localization at the SPB was not assayed in Mto1-truncation strains, because Alp4 remains present at the SPB even in *mto1*Δ cells, presumably via interaction with SPB protein Pcp1 on the nucleoplasmic face of the SPB [13].

Determination of Myp2-dependent Alp4 localization at the CAR used Alp4-GFP together with Rlc1-mCh. Alp4-GFP co-localized at the CAR with Rlc1-mCh in 29/44 wild-type cells (Rlc1 generally associates with the CAR earlier than Mto1 and Alp4). Alp4-GFP was not present at the CAR with Rlc1-mCh in *myp2*Δ cells (0/56 cells).

To measure Mto1-GFP signals at mSPBs in *cdc11*Δ and *sid4*Δ strains (**Fig. 4A, 4B**), the Mto1-GFP signal was measured within a 0.65 μm^2 circular region at the end of short spindles (< 6.5 µm in length, judging by mCherry-tubulin fluorescence). The signal from an identically-sized neighboring region was subtracted to calculate the net SPB-associated signal. 84 mSPBs in 8 *cdc11*Δ cells with multiple spindles, 22 mSPBs in 11 *cdc11*Δ cells with one spindle, 124 mSPBs in 14 *sid4*Δ cells with multiple spindles, and 20 mSPBs in 10 *sid4*Δ cells with one spindle were scored. Box in **Fig. 4B** shows median and interquartile range, and whiskers show interdecile range. Orange line shows upper bound (ninety-fifth percentile) from comparable measurements of non-SPB background areas, indicating that the "noise" in these measurements is relatively small.

To complement imaging of Mto1-GFP at iSPBs in *cdc11*Δ and *sid4*Δ multinucleate cells (**Fig. 4C**), we also treated these cells with MBC to depolymerize microtubules (as described above) and scored presence/absence of Mto1-GFP at iSPBs. Mto1-GFP remained at iSPBs after MBC treatment in multinucleate *sid4*Δ (118/123 SPBs, from 14 cells total) and *cdc11*Δ (58/58 SPBs, from 11 cells total).

To determine Mto1-GFP mSPB localization in temperature-sensitive SIN mutants (**Fig. 4E-4G, Fig. S4G**), cells were assayed after shift from 25°C to 36°C (in water bath) for 90-180 min, depending on the strain, and then imaged quickly on a microscope equipped with an objective heater (Bioptechs). Cut12-tdT, Sad1-dsRed, or RFP-Atb2 was used to indicate SPBs. In the experiment shown in **Fig. 4E**, Mto1-GFP was not observed at mSPBs in mononucleate mitotic *sid4-SA1* cells at 36°C (0/8 cells). Mto1-GFP was observed at mSPBs in *sid4-SA1* mutants at 25°C (data not shown). In the experiment shown in **Fig. 4F**, Mto1-GFP was observed at mSPBs in 3/3 wild-type cells at 36°C and in 7/7 *cdc11-123* cells at 25°C. In *cdc11-123* cells at 36°C (all mononucleate mitoses), Mto1-GFP was generally absent from SPBs, although very faint mSPB signals could be observed in 15/42 cells. In 11 of these 15 cells, the faint Mto1-GFP signal was at one SPB only. These faint signals are easily attributed to the rapid reversibility of the *cdc11-123* phenotype (e.g. during slide preparation; our unpublished observations). In the experiment shown in **Fig. 4G** Mto1-GFP was observed at 100/100 mSPBs in 25 multinucleate *cdc7-24* cells, at 48/48 mSPBs in 11 multinucleate *sid1-239* cells, and at 20/20 mSPBs in 5 multinucleate *sid2-250* cells.

Assays of microtubule re-growth after cold-shock (**Fig. S1J**) were as described previously [7]. Exponentially growing cells were chilled in ice water bath for 30 min and transferred to a pre-warmed flask and incubated at 32°C for the specified time before collection by filtration. Cells were fixed in methanol at -70°C and processed for anti-tubulin immunofluorescence, exactly as described previously [7, 51].

Astral Microtubules in cdc11Δ Mutants

The initial characterization of *cdc11*Δ mutants described astral MTs to frequently detach from SPBs during anaphase [30]. However, our analysis of microtubule dynamics in truncation mutants such as *mto1(1-1085)-GFP* shows that astral MT nucleation is tightly

correlated with Mto1 mSPB localization **(Fig. 1E, Fig. S1G, S1H)**. Therefore, our finding that Cdc11 is required for Mto1 mSPB localization would lead us to expect no astral MTs at all in *cdc11*Δ mutants. To clarify this apparent contradiction we reinvestigated astral MT nucleation in *cdc11*Δ mutants, using strains expressing GFP-tubulin at physiological concentrations [52], which may not have been used in earlier experiments [30].

We made two relevant observations: First, we found that *cdc11*Δ multinucleated cells are able to nucleate what appear to be astral MTs, but only in later stages of spindle elongation, and this corresponds with a return of Mto1-GFP to SPBs in *cdc11*Δ cells **(Fig. S4A, S4C, S4D)**. A likely explanation for the reappearance of Mto1-GFP at this stage is that, biochemically, these cells are already in the next interphase (i.e., with significantly reduced cyclin-dependent kinase activity), a stage when Cdc11 is no longer required for Mto1 SPB localization. The continued presence of spindles in these cells may also be due to lower-than-normal spindle disassembly rates, as the absence of a stable or well-organized CAR in SIN mutants [36] precludes the generation of robust PAAs [27], and PAAs may normally contribute to spindle disassembly by titrating away tubulin dimers; similarly slow spindle disassembly is observed in *mto1*Δ mutants [7]. Consistent with this view, we found that Mto1 localization and MT nucleation in *mto1(1-1085)-GFP cdc11*Δ double mutants were largely indistinguishable from *cdc11*Δ single mutants, even though *mto1(1-1085)-GFP* mutants lack the Mto1 mSPB localization signal **(Fig. S4B, S4D)**.

Second, although we did not observe astral MTs detaching from SPBs in *cdc11*Δ mutants, we found that both *cdc11*Δ and *mto1(1-1085)-GFP cdc11*Δ mutants often nucleate MTs very close to but not at SPBs as spindles were elongating **(Fig. S4D)**. This nucleation may occur from the surface of the nuclear envelope and/or the cell cortex, as it was also observed in mutants that lack any SPB-associated Mto1, such as *mto1(1-1051)-GFP* **(Fig. 1D, Fig. S1G, Movie S1)**. Depending on temporal resolution during imaging, this nucleation could be misinterpreted as released astral MTs [8, 53]. Thus overall, the earlier analysis of *cdc11*Δ mutants [30] can be reinterpreted in a manner that is consistent with our current results.

Biochemical Methods

To assay Mto1 levels in *mto1*-truncation strains, pelleted yeast cells were boiled for 5 minutes and then disrupted by bead-beating by using 0.5-mm zirconium beads in a buffer containing 50 mM Tris (pH 8.0), 150 mM NaCl, 1 mM EDTA, and 1 mM PMSF. Pulverized cells were boiled in Laemmli buffer without reducing agent for 5 min. Protein concentration of the cleared cell extract was measured by BCA assay and equal amounts loaded for SDS PAGE and Western blotting.

For co-immunoprecipitation experiments of Myp2-myc and Sid4-myc with GFP-VTD-Mto1 fragments (**Fig. 3E**), frozen yeast cells were disrupted by grinding with a mortar and pestle on dry ice, and then resuspended in 50mM HEPES (pH 7.5), 100mM NaCl, 1 mM EDTA, (0.1% Triton X-100), 1 mM phenylmethylsulfonyl fluoride (PMSF) and a protease inhibitor cocktail. Cleared cell extract was incubated with affinity-purified anti-GFP antibody immobilized on protein G Dynabeads for 60 min at 4°C. Dynabeads were washed four times with lysis buffer before heating at 70°C for 10 min in 2x SDS PAGE buffer, before use in SDS-PAGE and Western blotting. Co-immunoprecipitation experiments of Mto1 with GFP- and YFP-tagged proteins (**Fig. S3E**) were performed identically, except that the extract buffer was 50 mM HEPES (pH 7.5), 75 mM KCl, 1 mM MgCl2, 1 mM EGTA, 0.1% Triton X-100, 2 mM AEBSF, 1 mM benzamidine and a protease inhibitor cocktail.

Anti-Mto1 sheep polyclonal antibody, and TAT1 anti-tubulin [54] and 9E10 anti-myc [55] monoclonal antibodies were used as described previously [7]. Affinity-purified sheep anti-GFP antibodies were generated in-house. Secondary antibodies for immunofluorescence were Alexa-labeled (Invitrogen). Secondary antibodies for Western blotting were either horseradish peroxidase-labeled (GE Healthcare; Sigma-Aldrich; **Fig. S1C, Fig. S3E**), or IRDye-labeled (Licor Biosciences; **Fig. 3E**). HRP blots were developed by ECL. IRDye-labeled blots were imaged and analysed using an Odyssey imaging system (Licor Biosciences).

Yeast Two-Hybrid Assay

For yeast two-hybrid screening, a fragment containing Mto1 amino acids 769-1115 was generated by PCR and cloned into bait vector pBTM116, and screened in the strain *L40Δgal* against a meiotic *S. pombe* cDNA library cloned into pGAD424 (kind gift of T. Nakamura, Osaka City University, Japan), using conventional methods [56]. Smaller subfragments of Mto1(769-1115) and Cdc11(561-1045) were generated by PCR and subcloned into the same system. Semi-quantitative assays of betagalactosidase expression in two-hybrid strains were carried out using 2-nitrophenol-β-D-galactopyranoside as substrate. For two-hybrid assay of Mto1-Myp2 interaction, a Myp2 cDNA [57] was subcloned into pACT2.2gtwy (Addgene plasmid 11346, deposited by G. Caldwell).

Supplemental References

34. Campbell, R.E., Tour, O., Palmer, A.E., Steinbach, P.A., Baird, G.S., Zacharias, D.A., and Tsien, R.Y. (2002). A monomeric red fluorescent protein. Proc Natl Acad Sci U S A *99*, 7877-7882.

35. Wachtler, V., Rajagopalan, S., and Balasubramanian, M.K. (2003). Sterol-rich plasma membrane domains in the fission yeast Schizosaccharomyces pombe. J Cell Sci *116*, 867-874.

36. Hachet, O., and Simanis, V. (2008). Mid1p/anillin and the septation initiation network orchestrate contractile ring assembly for cytokinesis. Genes Dev *22*, 3205-3216.

37. Kobe, B., and Kajava, A.V. (2001). The leucine-rich repeat as a protein recognition motif. Curr Opin Struct Biol *11*, 725-732.

38. Bella, J., Hindle, K.L., McEwan, P.A., and Lovell, S.C. (2008). The leucine-rich repeat structure. Cell Mol Life Sci *65*, 2307-2333.

39. Moreno, S., Klar, A., and Nurse, P. (1991). Molecular analysis of the fission yeast *Schizosaccharomyces pombe*. Meth Enzymol *194*, 795-823.

40. Alfa, C., Fantes, P., Hymas, J., McLeod, M., and Warbrick, E. (1993). Experiments with Fission Yeast: a Laboratory Course Manual, (Cold Spring Harbor: Cold Spring Harbor Laboratory Press).

41. Bahler, J., Wu, J.Q., Longtine, M.S., Shah, N.G., McKenzie, A., 3rd, Steever, A.B., Wach, A., Philippsen, P., and Pringle, J.R. (1998). Heterologous modules for efficient and versatile PCR-based gene targeting in Schizosaccharomyces pombe. Yeast *14*, 943-951.

42. Shaner, N.C., Campbell, R.E., Steinbach, P.A., Giepmans, B.N., Palmer, A.E., and Tsien, R.Y. (2004). Improved monomeric red, orange and yellow fluorescent proteins derived from Discosoma sp. red fluorescent protein. Nat. Biotechnol. *22*, 1567-1572.

43. Snaith, H.A., Samejima, I., and Sawin, K.E. (2005). Multistep and multimode cortical anchoring of tea1p at cell tips in fission yeast. EMBO J. *24*, 3690-3699.
44. Basi, G., Schmid, E., and Maundrell, K. (1993). TATA box mutations in the Schizosaccharomyces pombe nmt1 promoter affect transcription efficiency but not the transcription start point or thiamine repressibility. Gene *123*, 131-136.
45. Sawin, K.E., and Nurse, P. (1996). Identification of fission yeast nuclear markers using random polypeptide fusions with green fluorescent protein. Proc Natl Acad Sci U S A *93*, 15146-15151.
46. Nagata, A., Igarashi, M., Jinno, S., Suto, K., and Okayama, H. (1991). An additional homolog of the fission yeast cdc25+ gene occurs in humans and is highly expressed in some cancer cells. New Biol *3*, 959-968.
47. Igarashi, M., Nagata, A., Jinno, S., Suto, K., and Okayama, H. (1991). Wee1(+)-like gene in human cells. Nature *353*, 80-83.
48. Snaith, H.A., and Sawin, K.E. (2003). Fission yeast mod5p regulates polarized growth through anchoring of tea1p at cell tips. Nature *423*, 647-651.
49. Sawin, K.E., and Snaith, H.A. (2004). Role of microtubules and tea1p in establishment and maintenance of fission yeast cell polarity. J. Cell Sci. *117*, 689-700.
50. Ge, W., and Balasubramanian, M.K. (2008). Pxl1p, a paxillin-related protein, stabilizes the actomyosin ring during cytokinesis in fission yeast. Mol Biol Cell *19*, 1680-1692.
51. Sawin, K.E., and Nurse, P. (1998). Regulation of cell polarity by microtubules in fission yeast. J Cell Biol *142*, 457-471.
52. Snaith, H.A., Anders, A., Samejima, I., and Sawin, K.E. (2010). New and old reagents for fluorescent-protein tagging of microtubules in fission yeast: experimental and critical evaluation Methods Cell Biol. *in press*.
53. Anders, A., Lourenco, P.C., and Sawin, K.E. (2006). Noncore components of the fission yeast gamma-tubulin complex. Mol. Biol. Cell *17*, 5075-5093.
54. Woods, A., Sherwin, T., Sasse, R., MacRae, T.H., Baines, A.J., and Gull, K. (1989). Definition of individual components within the cytoskeleton of *Trypanosoma brucei* by a library of monoclonal antibodies. J Cell Sci *93*, 491-500.
55. Evan, G.I., Lewis, G.K., Ramsay, G., and Bishop, J.M. (1985). Isolation of monoclonal antibodies specific for human c-myc proto-oncogene product. Mol Cell Biol *5*, 3610-3616.
56. Fromont-Racine, M., Rain, J.C., and Legrain, P. (2002). Building protein-protein networks by two-hybrid mating strategy. Methods Enzymol *350*, 513-524.
57. Matsuyama, A., Arai, R., Yashiroda, Y., Shirai, A., Kamata, A., Sekido, S., Kobayashi, Y., Hashimoto, A., Hamamoto, M., Hiraoka, Y., et al. (2006). ORFeome cloning and global analysis of protein localization in the fission yeast Schizosaccharomyces pombe. Nat Biotechnol *24*, 841-847.
