# Supplementary figures and images for: Fission Yeast Mto1 Regulates Diversity of Cytoplasmic Microtubule Organizing Centers
Source: Curr Biol. 2010 Nov 9;20(21):1959–65. doi: 10.1016/j.cub.2010.10.006 (PMC2989437; doi:10.1016/j.cub.2010.10.006)

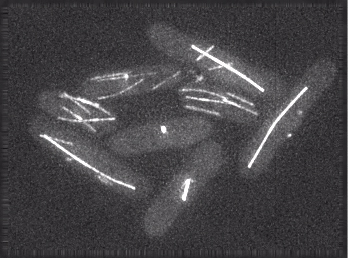

Supplement: Movie S1. Related to Figure 1. GFP-Tubulin (GFP-Atb2) Dynamics in mto1(1-1051)-GFP Cells — Wide-field images were taken every 15 s and deconvolved. Time compression is 150×. [file mmc3.jpg]

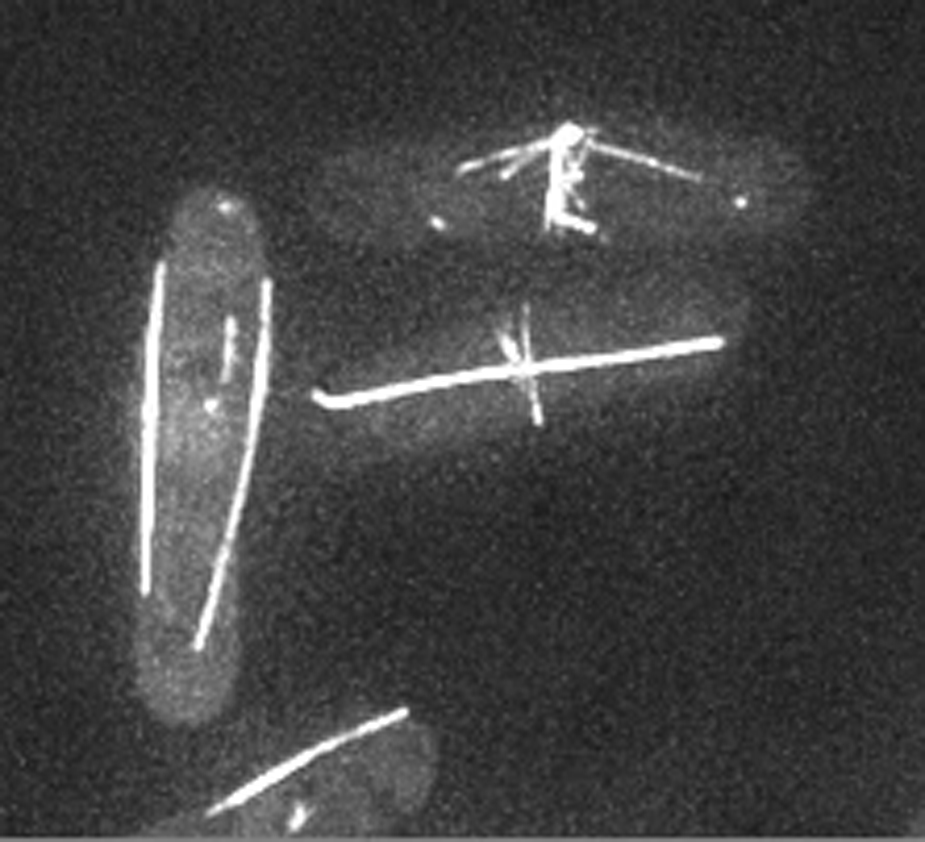

Supplement: Movie S2. Related to Figure 1. GFP-Tubulin (GFP-Atb2) Dynamics in mto1(1-1095)-GFP Cells — Wide-field images were taken every 15 s and deconvolved. Time compression is 150×. [file mmc4.jpg]
